# Association Between Serum Vitamin D Levels and Mortality in Children Receiving Chronic Dialysis: A Retrospective Cohort Study

**DOI:** 10.1002/hsr2.71804

**Published:** 2026-02-03

**Authors:** Sima Shamshiri Khamene, Izat Mohammad Khawajah, Mastaneh Moghtaderi

**Affiliations:** ^1^ School of Medicine Tehran University of Medical Sciences (TUMS) Tehran Iran; ^2^ Pediatric Chronic Kidney Disease Research Center, Gene, Cell & Tissue Research Institute, Children's Medical Center Tehran University of Medical Sciences Tehran Iran

## Abstract

**Background:**

Vitamin D deficiency is highly prevalent among children on regular dialysis, affecting approximately 90% of patients. This deficiency (serum 25‐hydroxyvitamin D < 50 nmol/L or 20 ng/mL) is associated with various complications, including skeletal problems, increased infection risk, arterial stiffness, vascular calcification, and higher cardiovascular mortality. Severe deficiency (< 30 nmol/L) particularly increases mortality risks.

**Method:**

In this cross‐sectional retrospective study, we examined 53 pediatric patients (28 boys, 25 girls) undergoing regular dialysis (hemodialysis and peritoneal dialysis) at a children's medical center from 2018 to 2020. The mean age was 8.21 years, with 71.7% aged 2–12 years, 20.8% adolescents, and 7.5% under 2 years. The mean vitamin D level was 23.51 ng/mL.

**Results:**

Results showed that 26.41% of patients died, with mortality analysis revealing a hazard ratio of 3.2 for patients with vitamin D levels below 15 ng/mL. The mortality rate was 64.7% in severe deficiency (< 15 ng/mL), 18.8% in moderate deficiency (15–30 ng/mL), while patients with sufficient levels (> 30 ng/mL) recorded no deaths. Additionally, 11.32% developed skeletal disorders, including two cases of spinal fracture. Vitamin D levels showed significant positive correlations with calcium (*r* = 0.6) and years under dialysis (*r* = 0.52) (*p* > 0.05). Associations were found between vitamin D levels and phosphorus, PTH, and mortality rates. However, no significant relationships were observed with dialysis frequency, age, weight, gender, underlying disease, dialysis type, or hypertension.

**Conclusion:**

In conclusion, children with end‐stage renal disease undergoing dialysis face increased risks of vitamin D deficiency due to impaired kidney function. This deficiency significantly impacts survival rates and contributes to poor outcomes. Regular monitoring and management of vitamin D levels are crucial for improving survival in pediatric dialysis patients.

## 1 Introduction

Vitamin D has multifunctional and also hormone‐like functions [1]. Most of the vitamin D in the body is produced in the skin (80%–90%) through exposure to UVB, and a small amount is derived from foods (10%–20%) [2]. Its hydroxylated form turns into 25‐hydroxy vitamin D(25‐OHD) in the liver, and the next hydroxylation takes part in the kidneys, turning it into active 1‐25hydroxy vitamin D (1‐25OH2D) [3, 4, 5]. Therefore, the main factors affecting serum vitamin D levels are sunlight, diet, age, gender, and weight. Main dietary sources of vitamin D are enriched milks, fish, and oral supplements. Skeletal hemostasis, reproductive system function especially in females, cardiovascular system, and calcium and phosphorus hemostasis depend on normal serum vitamin D (more than 30 ng/mL) [5, 6, 7, 8, 9, 10]. Decreased serum vitamin D may have some part in disorders such as depression, cancer, fractures, and impaired immune system function [10, 11]. Parathyroid hormone (PTH) and fibroblast growth factor 23 (FGF23) are the main regulators of vitamin D. Vitamin D deficiency is quite common in children with chronic kidney disease (CKD). It is one of the most common comorbidities in CKD patients, such as reduced glomerular filtration rate (GFR), proteinuria, and other diseases like diabetes [12, 13, 14, 15]. Impaired renal function is directly associated with secondary hyperparathyroidism [13], and the greater the GFR decrease, the greater the serum PTH level [5]. Advanced stages of CKD (stage 4–5) show more severe vitamin D deficiency compared to the moderate stages [3]. There is an elevated level of inflammatory factors and C Reactive Protein (CRP) in CKD patients and some evidence showed supplements can reduce these indices efficiently [15, 16]. Children on chronic dialysis programs probably have lower vitamin D levels because of impaired nutrition and dialysis itself [16, 17]. Lack of vitamin D causes hyperphosphatemia and hypocalcemia, resulting in increased PTH level and secondary hyperparathyroidism [18, 19]. FGF23 reduces PTH level in the early phase, but eventually both of them end up in the rise of it by some mechanisms [1, 20]. CKD children have a greater risk for vitamin D deficiency because they lack enough activity and don't get enough sunlight, and also, uremia itself reduces vitamin D synthesis. Therefore, supplements in the form of active vitamin D3 are suggested to be used in CKD stage 2–5 to prevent secondary hyperparathyroidism and its consequences [21]. Overall, up to 90% of patients who are routinely on dialysis programs are vitamin D deficient, which is associated with increased arterial stiffness and calcification of vasculature, stroke, and left ventricular hypertrophy, and also increased mortality due to cardiovascular accidents [1, 12]. The purpose of this study was to review and assess children on regular dialysis programs and evaluate their serum vitamin D level and its relationship with calcium, phosphorus, serum PTH, and mortality in these patients.

## 2 Materials and Methods

The study was designed as a retrospective observational cross‐sectional study, and no new intervention was performed. All data from this study were collected retrospectively. All patients undergoing regular hemodialysis and peritoneal dialysis from 2018 to 2020 (53 patients) enrolled in the study. Patients with underlying non‐renal diseases such as hypertension, adrenal hyperplasia, coarctation of the aorta, and pheochromocytoma were excluded from the study. The variable of interest was age, gender, body mass index (BMI), drugs, type of dialysis, hypertension, calcium (Ca), phosphorus(P), and vitamin D level, PTH and CRP and urine residue, as well as diet compliance were evaluated every 1–3 months. Arterial hypertension is defined by a sustained rise of blood pressure in 95% of recorded charts of BP. The *χ* ^2^ test was used to analyze the categorical variables. Statistical inferences related to associations between the serum 25(OH)D level and the outcomes were analyzed using the multivariate Cox proportional hazards regression analyses. *p* values more than 0.05 were considered statistically insignificant. All statistical analyses were done using statistical package for the social sciences (SPSS) Statistics 21 and analyzed to find any meaningful relations.

## 3 Results

This comprehensive study analyzed 53 pediatric dialysis patients, consisting of 28 boys and 25 girls. The demographic distribution revealed an average age of 8.21 months with a median of 9 years. The age categories were distinctly represented, with adolescents (over 12 years) comprising 8.2% of the population, children between 2 and 12 years representing the majority at 71.7%, and infants under 2 years accounting for 7.5% of the study population.

The assessment of residual kidney function revealed important distinctions among the patients. Thirty patients (56.7%) demonstrated no residual kidney function, while 23 patients (43.3%) maintained varying degrees of residual function. The metabolic status of the patients showed significant variations, with 32 patients (60%) presenting with metabolic acidosis, a single patient (1.8%) exhibiting metabolic alkalosis, and 20 patients (38.2%) maintaining normal acid‐base balance.

Laboratory analyses yielded crucial insights into vitamin D metabolism and related parameters. The mean serum vitamin D level was 23.51 ng/mL (median 22), displaying a positively skewed distribution that suggested a tendency toward deficiency in the study population. Parathyroid hormone levels demonstrated considerable variation, with a mean PTH level of 411.57, a median of 290, and a mode of 110, indicating diverse patterns of secondary hyperparathyroidism among the patients.

Statistical analyses revealed significant relationships between various parameters. Vitamin D levels showed a moderate positive correlation with calcium levels (correlation coefficient 0.6, *p* < 0.05) and duration of dialysis (correlation coefficient 0.52, *p* < 0.05). Through linear regression analysis, it was determined that vitamin D levels explained 12% of calcium variations (adjusted *R*² = 0.12). The duration of dialysis demonstrated a weak but significant correlation with calcium levels (correlation coefficient 0.2), with dialysis duration explaining 5% of serum calcium variations.

**Figure 1:**
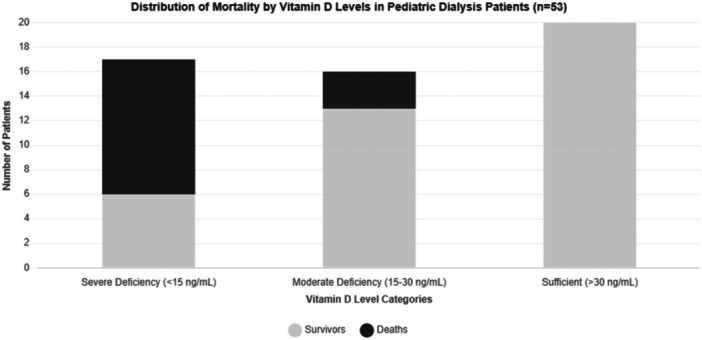
Distribution of mortality by vitamin D level.

The mortality analysis revealed particularly striking findings. During the study period, 14 deaths were recorded, with statistical analysis demonstrating a highly significant association between vitamin D levels and mortality (*p *< 0.001). Patients with vitamin D levels below 15 ng/mL faced a hazard ratio of 3.2, indicating more than three times the mortality risk compared to those with higher vitamin D levels. The mortality pattern demonstrated clear stratification based on vitamin D levels, with severely deficient patients (< 15 ng/mL) experiencing a 64.7% mortality rate, moderately deficient patients (15–30 ng/mL) showing an 18.8% mortality rate, while remarkably, patients maintaining sufficient vitamin D levels (> 30 ng/mL) recorded no deaths during the study period (Figure 1). The 24‐month survival analysis further reinforced these findings, demonstrating a clear gradient in survival rates. Patients with sufficient vitamin D levels maintained 100% survival, while those with severe deficiency showed only 35.3% survival. Notably, vitamin D levels below 15 ng/mL during summer months were particularly associated with increased mortality risk, establishing this threshold as a critical marker for clinical assessment.

Comparative analysis between survivors and non‐survivors revealed no significant differences in several clinical parameters, including sex, dialysis frequency, weight, BMI, and CRP levels, suggesting that these factors may not be primary determinants of mortality in this population. Additionally, no significant correlations were identified between vitamin D levels and phosphorus, PTH, dialysis frequency, age, or weight (*p* > 0.05), suggesting independent regulatory mechanisms for these parameters.

The relationship between vitamin D levels and mortality remained robust and significant even after adjusting for potential confounding factors, establishing vitamin D status as an independent predictor of mortality risk. The identification of 15 ng/mL as a critical threshold for increased mortality risk provides an important clinical benchmark for risk assessment and intervention. These findings strongly suggest that vitamin D levels serve as a reliable prognostic indicator in pediatric dialysis patients, offering valuable guidance for clinical management and therapeutic intervention strategies. Figure 1 shows the distribution of mortality by vitamin D levels. Table 1 demonstrates the demographic features in the study population, Table 2 shows the qualitative variables in the study population, and Table 3 presents the correlation of independent variables with vitamin D in dialysis in our patients.

**Table 1:** Demographic features in the study population.

	Age (year)	Weight (kg)	Vitamin D (ng/mL)	Ca	P	PTH	Number of dialysis/weak	Years on dialysis
Mean	8.21	26.64	33.51	8.98	6.18	411.57	2.74	3.17
Median	9	26	33	9	6	290	3	2
Mode	5	12	22	9	4.30	110	3	1
Std. deviation	4.316	17.009	18.421	1.05	1.81	354.37	0.44	2.73
Variance	18.62	289.31	339.33	1.11	3.30	125583.28	0.19	7.46
Range	14	73	80	4.20	6.80	1403	1	10
Minimum	1	4	4	6.80	3.30	27	2	1
Maximum	15	77	84	11	10.10	1430	3	11
Percentile								
25	5	12	20	8.4	4.5	128.5	2	1
50	9	26	33	9	6	290	3	2
75	12	36	43.5	9.8	7.4	581.5	3	4

Abbreviations: Ca, Calcium; Ph, phosphorus; PTH, parathyroid hormone, if multiple modes exist then the smallest value is shown for mode.

**Table 2:** Qualitative variables in the study population.

		Frequency	Percent	Valid percent	Cumulative percent
PUV + neurogenic bladder		16	30.2	30.2	30.2
Renal dysplasia		8	15.1	15.1	45.3
RPGN/FSGS		8	15.1	15.1	60.4
CHD, ARPKD, interstitial nephritis		21	39.6	39.6	100
Renal residue	Yes	21	39.6	39.6	39.6
	No	32	60.4	60.4	100
Hemodialysis		30	56.6	56.6	56.6
Peritoneal dialysis		23	43.4	43.4	100

Abbreviations: CHD, congenital heart disease; FSGS, focal segmental glomerulosclerosis; PUV, posterior urethral valves, RPGN, rapidly progressive glomerulonephritis.

**Table 3:** Correlation of independent variables with vitamin D in dialysis patients.

	HTN	Mean	Std. deviation	*p* value
Hemoglobin	Yes	10.9977	1.02627	0.05
	No	12.1750	1.76048	
PTH	Yes	164.100	87.6654	0.991
	No	165.250	139.2465	
Vitamin D	Yes	23.516	8.5280	0.808
	No	41.333	12.5628	
Time of hemodialysis per day	Yes	2.853	0.5524	0.155
	No	2.588	0.5073	
Number of dialysis per week	Yes	2.941	0.5557	0.259
	No	3.176	0.6359	

## 4 Discussion

In this study of 53 pediatric dialysis patients, our most significant finding was the strong association between vitamin D levels and mortality, with patients having vitamin D levels below 15 ng/mL showing a hazard ratio of 3.2. This relationship was clearly demonstrated in mortality rates, where patients with severe vitamin D deficiency (< 15 ng/mL) experienced a 64.7% mortality rate, those with moderate deficiency (15–30 ng/mL) showed an 18.8% mortality rate, while remarkably, patients with sufficient vitamin D levels (> 30 ng/mL) recorded no deaths. Our analyses also revealed significant positive correlations between vitamin D levels and both calcium levels and duration of dialysis, though no significant correlations were found with other clinical parameters.

Solarin and his colleagues conducted a retrospective investigation in 2019 that focused on the vitamin D status of children with moderate to severe chronic kidney disease. The research involved 46 children and adolescents below the age of 18, including 22 girls and 24 boys [16]. The findings indicated that while low vitamin D levels were linked to older age, there was no significant inverse correlation between vitamin D and PTH. In our study also, no significant association was observed between vitamin D and the age of children. The Solarin study revealed that there was no correlation between gender and vitamin D levels, and those undergoing peritoneal dialysis exhibited lower vitamin D levels. Our study had similar results. In our study mortality rate had a significant relationship with peritoneal dialysis patients and low serum level of vitamin D. In a cross‐sectional study published in 2017 by Paulo Coccia and his colleagues, they studied 32 healthy control and 135 cases of CKD children and adolescents under 19 years old, they found Vitamin D deficiency is prevalent in 12.5% of controls and 25% of patients. Patients with vitamin D deficiency had higher PTH levels and phosphate levels [22]. In our study, univariate analyses showed vitamin D level is directly associated with calcium and phosphorus (*p* > 0.05), but there was no significant association with age, sex, weight, underlying disease, urinary residue, and the number of dialyses per week (*p* < 0.05). In a case‐control study published by Doaa M. Youssef and her colleagues on 27 ESRD cases and 20 controls in 2010, they found no significant relationship between age, disease duration, dialysis duration, and PTH between the subgroups of ESRD [23]. Similar to our findings, where calcitriol supplementation in dialysis patients showed no significant effect on mortality and spinal fracture rates, only one study has demonstrated improved survival rates with calcitriol treatment in hemodialysis patients [17]. Another cross‐sectional study by Debora R. Stein and her colleagues on 100 CKD patients, including 60 boys and 40 girls in 2012 found a high prevalence of hyperparathyroidism in the early stages of disease and a significant relationship between vitamin D and PTH. Also, this study showed that improving vitamin D levels is effective in controlling of hyperparathyroidism [24]. Unlike our study, no significant relationship between vitamin D and PTH was observed. Based on Spearman's correlation analysis there was a weak relationship between calcium and years of dialysis with vitamin D (*p* < 0.05). In cross‐sectional study done by H. Taskapan and his colleagues in 2006, on 273 children undergoing peritoneal dialysis (123 girls and 150 boys) they did not find any significant relation between the level of 25(OH)D3 and the gender of the patients [25]. Also, they report an inverse correlation between the level of 25(OH)D3, age and the presence of diabetes, and a direct correlation of calcium and 1,25(OH)2D3 was reported. In our study, we find a significant correlation between serum vitamin D and calcium, but there is no significant relation with the gender of the patients. Maryanne Machado da Silva Canhos and colleagues showed annual average vitamin D levels < 23.1 ng/mL were associated with higher all‐cause mortality [26]. Regardless of the confounding variables evaluated, in our study serum vitamin D less than 15 ng/ml was associated with higher mortality rate although some studies suggest serum level of 25(OH)D below 23.6 ng/mL in summer is associated with higher mortality rates [26].

## 5 Implication for Practice

According to our study, managing vitamin D supplementation in children on dialysis can follow standard WHO (KDIGO) guidelines, similar to the approach used for healthy children, regardless of their serum vitamin D levels. We observed that children with CKD who maintain residual kidney function typically demonstrate more favorable vitamin D levels, which can be attributed to their remaining functional renal tissue continuing to participate in vitamin D metabolism. For optimal care of children on regular dialysis programs, consistent monitoring of vitamin D, calcium, phosphorus, and PTH levels is essential. This vigilant monitoring approach plays a crucial role in preventing renal osteodystrophy, reducing the risk of bone fractures, avoiding cardiovascular complications, and ultimately lowering both morbidity and mortality rates in this vulnerable population.

## Author Contributions

**Sima Shamshiri Khamene:** investigation, methodology, software, and formal analysis. **Izat Mohammad Khawajah:** investigation, methodology, software, and formal analysis. **Mastaneh Moghtaderi:** conceptualization, investigation, writing – original draft, methodology, validation, visualization, writing – review and editing, software, formal analysis, project administration, data curation, supervision, and resources.

## Funding

The authors received no specific funding for this work.

## Ethics Statement

The method was approved in compliance with scientific and ethical standards. All procedures were performed in line with the relevant guidelines and regulations. The Medical Ethics Committee of Tehran University of Medical Sciences approved this study.

## Informed Consent

As our patients were under 16 years old, all the parents or guardians were informed and signed the informed consent form.

## Conflicts of Interest

The authors declare no conflicts of interest.

## Transparency Statement

The corresponding author, Mastaneh Moghtaderi, affirms that this manuscript is an honest, accurate, and transparent account of the study being reported; that no important aspects of the study have been omitted; and that any discrepancies from the study as planned (and, if relevant, registered) have been explained.

## Data Availability

The data supporting this study's findings are available on request from the corresponding author. The data sets created during the current study are not publicly accessible due to the possibility of compromising individuals' privacy.
